# Preliminary results of proton radiotherapy for pediatric rhabdomyosarcoma: a multi‐institutional study in Japan

**DOI:** 10.1002/cam4.1464

**Published:** 2018-03-31

**Authors:** Masashi Mizumoto, Shigeyuki Murayama, Tetsuo Akimoto, Yusuke Demizu, Takashi Fukushima, Yuji Ishida, Yoshiko Oshiro, Haruko Numajiri, Hiroshi Fuji, Toshiyuki Okumura, Hiroki Shirato, Hideyuki Sakurai

**Affiliations:** ^1^ Department of Radiation Oncology University of Tsukuba Tsukuba Ibaraki Japan; ^2^ Division of Proton Therapy Shizuoka Cancer Center Hospital Nagaizumi Shizuoka Japan; ^3^ Division of Radiation Oncology and Particle Therapy National Cancer Center Hospital East Kashiwa Chiba Japan; ^4^ Department of Radiology Hyogo Ion Beam Medical Center Tatsuno Hyogo Japan; ^5^ Department of Child Health University of Tsukuba Tsukuba Ibaraki Japan; ^6^ Division of Pediatrics Shizuoka Cancer Center Hospital Nagaizumi Shizuoka Japan; ^7^ Department of Radiology National Center for Child Health and Development Tokyo Japan; ^8^ Department of Radiation Oncology Hokkaido University Hospital Sapporo Japan

**Keywords:** Outcomes, pediatric, proton radiotherapy, rhabdomyosarcoma, toxicity

## Abstract

To evaluate preliminary results of proton radiotherapy (PRT) for pediatric patients with rhabdomyosarcoma (RMS). From 1987 to 2014, PRT was conducted as initial radiotherapy in 55 patients (35 males, 20 females, median age 5 years, range 0–19) with RMS at four institutes in Japan. Thirty‐one, 18, and six patients had embryonal, alveolar, and other RMS, respectively. One, 11, 37, and six patients were in IRSG groups I, II, III, and IV, respectively, and the COG risk group was low, intermediate, and high for nine, 39, and seven patients, respectively. The irradiation dose was 36–60 GyE (median: 50.4 GyE). The median follow‐up period was 24.5 months (range: 1.5–320.3). The 1‐ and 2‐year overall survival rates were 91.9% (95% CI: 84.3–99.5%) and 84.8% (95% CI 75.2–94.3%), respectively, and these rates were 100% and 100%, 97.1% and 90.1%, and 57.1% and 42.9% for COG low‐, intermediate‐, and high‐risk groups, respectively. There were 153 adverse events of Grade ≥3, including 141 hematologic toxicities in 48 patients (87%) and 12 radiation‐induced toxicities in nine patients (16%). Proton‐specific toxicity was not observed. PRT has the same treatment effect as photon radiotherapy with tolerable acute radiation‐induced toxicity.

## Introduction

Rhabdomyosarcoma (RMS) is the most common malignancy in childhood soft tissue sarcoma. Multimodal treatment is essential for RMS, and radiotherapy plays an important role. The 5‐year failure‐free survival (FFS) rates are 90%, 60–70%, and 20–30% for low‐, intermediate‐, and high‐risk patients, respectively 1, 2, 3, 4, 5. However, toxicities of radiotherapy are a significant problem in pediatric patients 6. This is a particular concern in RMS because the common sites are the head and neck and parameningeal lesions, for which bone retardation and growth hormone deficiency after radiotherapy are severe problems 7. Also, secondary cancer is an important issue in children. Proton radiotherapy (PRT) is likely to reduce these toxicities compared to photon radiotherapy, and dosimetric advantages of PRT have been suggested for treatment of RMS 8, 9. However, there are few clinical studies of PRT for RMS. Here, we evaluated preliminary results of PRT for RMS in a multicenter study in Japan.

## Materials and Methods

A retrospective observational study of PRT was performed in 71 pediatric patients (aged <20 years old) with primary RMS treated at four institutions in Japan from 1987 to 2014. Fifteen patients who received PRT for recurrent or secondary RMS were excluded from the study. One patient with multiple spinal metastases during PRT, in whom the treatment strategy was changed from local irradiation with PRT to craniospinal irradiation with photon radiotherapy, was also excluded, leaving 55 patients for analysis. The study was approved by each Institutional Review Board. We have previously reported an overview of this study 10, 11, in which we mainly analyzed overall survival and late toxicities of all patients and long‐term survivors. Multiple tumor types were also included in the analysis, and so details for specific tumors were unclear. To properly define the treatment outcomes of PRT for RMS, we performed a secondary analysis focused only on patients with RMS.

### Statistical analysis

The primary endpoints of this study are overall survival (OS), progression‐free survival (PFS), and local control rate (LCR) estimated using the Kaplan–Meier method, and the secondary endpoint is acute adverse effects of PRT. OS, PFS, and LCR were measured from the start of PRT until the respective event, including death for OS and tumor progression at the primary site for LCR. A log‐rank test was used to evaluate differences in OS, PFS, and LCR among three risk groups based on COG criteria at the time the study protocol was written. All analyses were performed with SPSS ver. 11.0 (SPSS Inc., Chicago, IL). Toxicities were graded using the Common Terminology Criteria for Adverse Events ver. 3.0.

## Results

A total of 55 patients (35 males and 20 females) (Table 1) had a median age at treatment of 5 years old (range: 0–19). Thirty‐one patients had embryonal RMS, 18 had alveolar RMS, and six had another form of RMS. The irradiated sites were the head and neck (*n* = 37), parameningeal sites (*n* = 3), prostate (*n* = 8), and others (*n* = 7). One, 11, 37, and six patients were classified into IRSG surgicopathologic groups I, II, III, and IV, respectively, and the COG risk group was low, intermediate, and high for nine, 39, and seven patients, respectively. The irradiation dose ranged from 36 to 60 GyE (median: 50.4 GyE). Surgical resection before PRT was performed in 41 patients (75%), and 53 patients (96%) received chemotherapy: pre‐PRT, pre‐ and during PRT, and only during PRT in 17, 34, and two patients, respectively. The median follow‐up period in all 55 patients was 24.5 months (range: 1.5–320.3 months), and 11 patients were followed up for ≥5 years.

**Table 1:** Patients and PBT characteristics (*n* = 55)

Item	Value
Age, years (median)	0–19 (5)
Sex (male/female)	35/20
Histology	
Embryonal	31
Alveolar	18
Others	6
Irradiation site	
Head and neck	37
Parameningeal	3
Prostate	8
Others	7
Group	
I	1
II	11
III	37
IV	6
Risk group	
Low	9
Intermediate	39
High	7
PBT dose, GyE	
Median	50.4
Range	36.0–60.0

**Figure 1:**
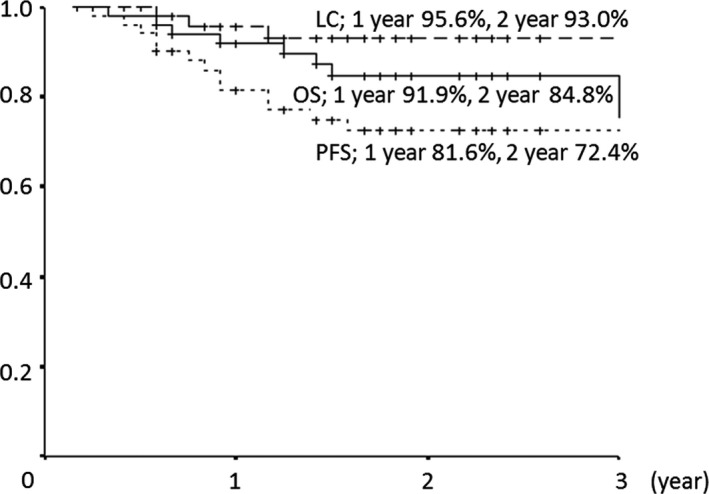
Overall survival (OS), progression‐free survival (PFS), and local control (LC) rates in all patients.

**Figure 2:**
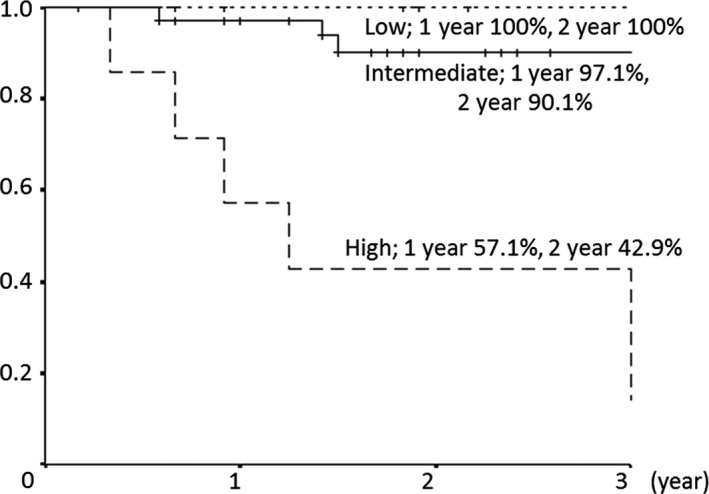
Overall survival rate according to COG risk group.

At final follow‐up, the 1‐ and 2‐year OS rates were 91.9% (95% CI: 84.3–99.5%) and 84.8% (95% CI: 75.2–94.3%), respectively, and the 1‐ and 2‐year PFS rates were 81.6% (95% CI: 70.7–92.5%) and 72.4% (95% CI: 59.6–85.3%), respectively. Nine patients died: seven due to tumor progression, one due to secondary cancer, and one due to a non‐tumor‐related cause. Thirteen patients had recurrence, including five with local recurrence and eight with distant metastases. The 1‐ and 2‐year LCRs were 95.6% (95% CI: 89.6–100%) and 93.0% (95% CI: 85.3–100%), respectively (Fig. 1). In the COG low‐, intermediate‐, and high‐risk groups, the 1‐ and 2‐year LCRs were 100% and 100%, 96.8% and 96.8%, and 80.0% and 53.3%, respectively. LCRs were significantly poor at high‐risk group (*P* = 0.009). The 1‐ and 2‐year OS rates were 100% and 100%, 97.1% and 90.1%, and 57.1% and 42.9%, respectively (Fig. 2). OS was significantly poor at high‐risk group (*P* = 0.001). And the 1‐ and 2‐year PFS rates were 85.7% and 85.7%, 88.2% and 81.8%, and 42.9% and 14.3%, respectively. PFS was also significantly poor at high‐risk group (*P* = 0.001).

At first, we focused on acute toxicities (Table 2). There were a total of 153 acute toxicities of Grade ≥3: 141 hematologic toxicities in 48 patients (87%) and 12 radiation‐induced toxicities (including mucositis and dermatitis) in nine patients (16%). At the start of PRT, 60 hematologic toxicities were present due to previous treatment. Changes in hematologic toxicities from the start of PRT to maximum toxicity during PRT are that 23 (42%), 34 (62%), and 33 (60%) patients had exacerbation of anemia, decreased white blood cells, and decreased neutrophil count, respectively. In 19 patients who received PRT without concurrent chemotherapy, exacerbation of these events occurred in four (21%), nine (47%), and seven (37%), respectively. For patients who received concurrent chemotherapy, similar exacerbation occurred in 19 (53%), 25 (69%), and 26 (72%), respectively.

**Table 2:** Acute toxicities of Grade 3 or higher

Toxicity	Grade 3	Grade 4
Appetite loss	2	0
Dermatitis	3	0
Mucositis	5	0
Anemia	18	13
White blood cell decreased	5	39
Neutrophil count decreased	5	35
Plate count decreased	9	12
Electrolyte abnormality	2	1
GOT/GPT increased	2	0
Blood bilirubin increased	0	0
Others	2	0

Others: Infection.

As for late toxicities, eight patients experienced nine Grade 2 late toxicities. Six were deformity, and three were others (chronic otitis, growth hormone deficiency, and hearing impairment). Grade 3 or more late toxicities were not occurred. All Grade 2 late toxicities were occurred in the patients with head and neck or parameningeal tumor, and seven of 10 patients, who survive 5 years or more, experienced Grade ≥2 late toxicities.

## Discussion

**Figure 3:**
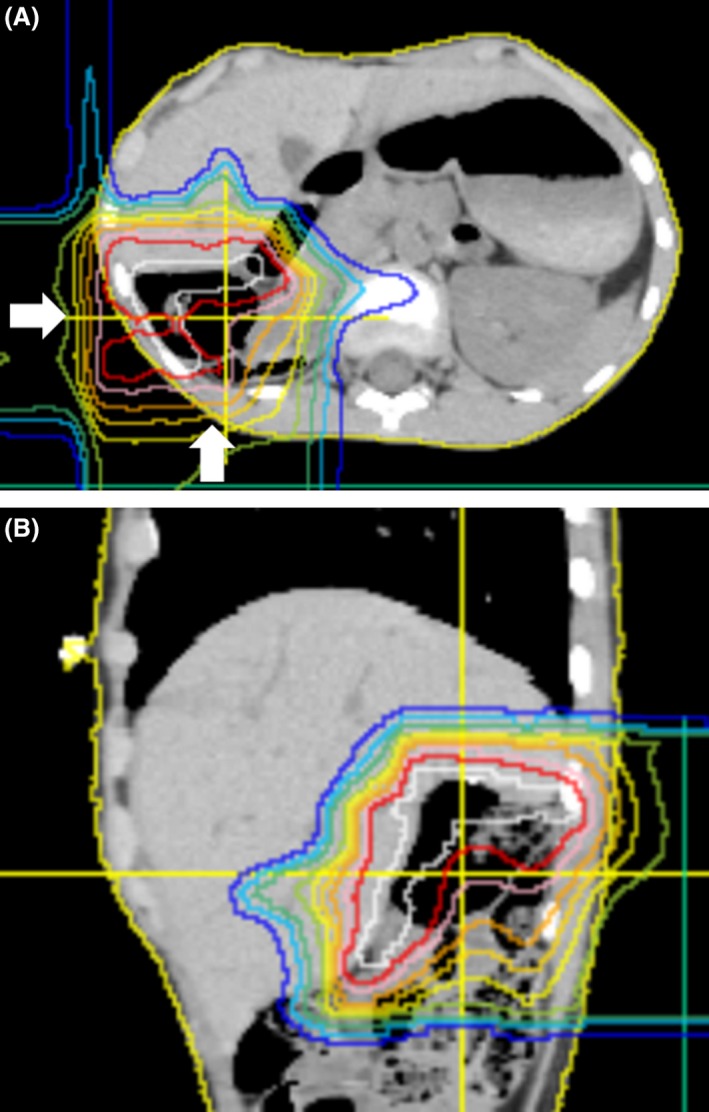
Dose distribution for abdomen rhabdomyosarcoma (embryonal type, group II). Proton radiotherapy of 41.4 GyE in 23 fractions was performed for tumor bed. Outside the blue line was not irradiated. Most part of normal liver was not irradiated (B, sagital view). Proton beam was irradiated from behind and right side (A, white arrow).

In this analysis, the short median follow‐up period allowed evaluation of only short‐term treatment outcomes and toxicities. In the low‐, intermediate‐, and high‐risk groups, the 2‐year OS rates were 100%, 90%, and 42.9%, and the 2‐year PFS rates were 85.7%, 81.8%, and 14.3%, respectively. In the COG trial, the respective 2‐year OS/FFS rates were 95–100%/85–90%, 80–90%/70–80%, and 40–50%/20–30% (with chemotherapy) 1, 2, 3, 4, 5. Our results are similar to these findings. In Japan, PRT is mainly used to reduce late toxicity, and treatment for RMS is normally based on photon radiotherapy [12, 13, Fig. 3]. Our results indicate that PRT can achieve the same treatment outcomes as photon radiotherapy. Two recent reports of PRT for RMS also showed similar results. In preliminary results of a phase II trial of PRT for RMS, Ladra et al. 14 found 2‐year OS rates of 100% and about 80% for low‐ and intermediate‐risk patients, respectively. In the largest clinical study of PRT performed to date, in 83 patients (risk: low 20, intermediate 52, high 11), Leiser et al. 15 found 2‐year OS rates of 80‐90% after PRT. Both reports also concluded that PRT gives similar outcomes compared to treatment with photon radiation.

Our analysis showed a low rate of radiation‐related acute toxicity of Grade ≥3 of 16% and good recovery after PRT. Most patients (87%) had hematologic toxicities of Grade ≥3, and it is difficult to determine which treatment (chemotherapy, surgery, or radiotherapy) caused this toxicity. However, the risk of exacerbation of hematologic toxicities during PRT was twice as high in patients receiving concurrent chemotherapy (anemia 53%, white blood cells 69%, neutrophils 72%) than those treated with nonconcurrent chemotherapy (anemia 21%, white blood cells 47%, neutrophils 37%). Arndt et al. 3 found rates of Grade 3 or 4 acute toxicity in treatment of intermediate‐risk RMS of about 80% (neutropenia) and 50% (anemia). In our analysis, the schedule of chemotherapy was unknown, but the risk of acute hematologic toxicity looks similar. In a recent study of Grade 3 and Grade 4 acute toxicity after irradiation in children, Pixberg et al. 16 reported acute toxicity rates of 63.8% in bone marrow, 7.6% in skin, and 7.6% in mucosa. These rates are also similar to our data for skin three of 55 (5.5%) and mucosa five of 55 (9.1%). Additionally, in multivariate analysis, Pixberg et al. 16 showed that concomitant chemotherapy was a risk factor for acute toxicity, and our results show a similar trend. In patients with head and neck RMS treated by PRT, Ladra et al. showed acute toxicity rates of 6–8% for Grade 3 dermatitis and 3% for mucositis, with a total Grade 3 rate of 17%, which is close to our rate of 16%. At this time, follow‐up period was still short, but all Grade 2 or more late toxicities occurred for head and neck or parameningeal tumor. This looks same tendency that we reported previously. The most likely benefit of PRT is to reduce late toxicity, but obtaining these data will require longer follow‐up in more patients. However, our results indicate that PRT can achieve the same short‐term treatment effect as photon radiotherapy with tolerable acute radiation‐induced toxicity.

## Conflict of Interest

Dr. Hiroki Shirato has received donations from Hitachi Ltd., Shimadzu Corp., and Jokoh. All other authors have no financial support or relationships to declare.
